# Health policy and regulations in Seychelles – barriers and opportunities for oral health inclusion

**DOI:** 10.1186/s12903-024-04321-7

**Published:** 2024-05-24

**Authors:** C. Y. S. Noshir, P. Brijlal

**Affiliations:** Faculty of Dentistry, Department of Oral Hygiene, University of Western Cape, Cape Town, South Africa

**Keywords:** Seychelles, Oral health policy, Public health policy, Service delivery, Oral health outcomes, Inclusion of oral health

## Abstract

**Background:**

Successful and inclusive policies that embrace oral health as part of the health agenda have the potential to alleviate the burden of oral diseases and to promote dental public health. This study aimed to understand the factors influencing the inclusion of oral health in health and public policy and regulations in the Seychelles. The barriers and opportunities for inclusion / non-inclusion as well as the impact thereof were explored.

**Methodology:**

A qualitative approach was adopted using document analysis and interviews as data collection strategies to allow for a complete analysis of the research problem. Using a purposive sampling approach, individual face to face interviews were conducted with patients, dental staff and representatives of the upper management. Policy and related oral health statistical documents were reviewed to ascertain how oral health was located and implemented from a national to a district level. Thematic analysis and content analysis were used to analyse and interpret the qualitative data.

**Results:**

The study provided insight on how oral health is contextualised in the Seychelles and how public policy and strategic documents influences the oral health outcomes. There is fragmentation in how the health and oral health agendas are managed and it is coupled with a severe lack of involvement and commitment to address the latter.

**Conclusion:**

Oral health needs to be integrated in all relevant policies and public health programmes as part of the broader national NCDs in Seychelles in order reduce the incidence of oral diseases in the population.

## Introduction

Oral diseases are a global health problem that impacts on quality of life and places an avertable burden on the healthcare system and the economy [1]. Public health policies which have the potential to intensify or reduce the social disparity in health aim to foreground important health issues, facilitate monitoring and, inform service provision through solution driven strategies [1, 2]. However minimal attention has been given to the inclusion and integration of oral health in public health policies. For decades, policy declarations [3, 4] and research [1, 5–9] on oral health inclusion has emphasised that successful and inclusive health policies can decrease the burden of oral diseases and promote dental public health.

Despite this appeal, the political attention to the oral health burden remains low from some government sectors and has often been overlooked in national and global health plans and surveys that should inform country needs [10–12]. This neglect may be due to factors such as lack of insight or agreement on the severity of the problem at hand and possible lack of resources and viable solutions [5, 10, 11].

Whilst many countries have put in place general policies to address oral health actions, there has been differing approaches between nations, and also within countries at national and regional levels [12, 13]. There are also differences in how policies are framed with some focusing attention on isolated strategies instead of fully integrated oral health plans [12, 13]. It has been well documented that the low representation of oral health on the global agenda has generally resulted in a poor allocation of funding [1], and lacking in clear guidelines for the translation of policies into practice. These problems may be attributable to factors previously recognised [9] such as inconsistency and fragmentation in the integration of health / oral health policy initiatives, and the lack of evidence of policy implementation and evaluation which still has relevance today [1]. Following continued efforts, the World Health Organisation, in its global oral health status report towards universal health coverage for oral health by 2030 [1] maintains that common risks such as smoking, harmful alcohol use and unhealthy diet that are shared by oral health and systemic health warrants the need for integrated policy guidelines. These guidelines are recommended to address the determinants of poor oral health, common risks, and ensure that oral health is available to all without hardship [1]. However, there are countries [6], that are still challenged with the lack of oral health prioritisation, and inclusion in health policies. This study focuses on the Seychelles which has similar challenges in the way that policy and regulation influences oral health care.

## Background

Seychelles, which has a population of 107,000, is a WHO member state. Primary health care in the Seychelles is free of charge as per the Constitution [14]. The Health Ministry is headed by the Minister alongside the Principal Secretary and is accountable to create health sector policy, planning, monitoring and evaluation [14]. The Ministry also monitors the operations of health strategies provided by three public bodies managing health care training and delivery. That is, the Health Care Agency (HCA), the Public Health Authority and the National Institute of Health and Social Studies (NIHSS). The HCA is an independent agency which brings about the provision of primary, secondary and tertiary health care. The Oral Health Services (OHS) is a division under the HCA. Majority of the Seychelles population access oral health services provided by the OHS [14].

Results of past surveys show that oral diseases remain a burden in Seychelles [14–18]. An early study in 1993 using DMFS scores reported an index of 58.5 in Seychellois adults aged 50 and above [15]. In 2019, the prevalence of untreated caries of deciduous teeth in children was recorded at 43.5% whilst in permanent teeth it was 26.4% [18]. Prevalence of severe periodontal disease was reported at 11.7% and 5% prevalence of edentulism [18]. Furthermore, statistical data shows that majority of patients accessing public dental facilities are coming on an emergency basis for the relief of pain and without an appointment [15, 18]. In addition, the majority of oral health personnel in Seychelles are employed by the government with the ratio of oral health care personnel to population in Seychelles being lower compared to Africa but higher than industrialized countries. For example, in 2016 and 2017 the ratio of dentist to population in Seychelles was 1:4246 and 1:2785 respectively. In Africa and industrialized countries, the ratio is 1:150000 and 1:2000 respectively [19].

The health system in Seychelles is guided by different parts of policy and strategic plans that contribute to and expand on the National Health Policy (2016) [20], National School Nutrition Policy (2008) [21], Seychelles National Health Strategic Plan (2016–2020) [22], and the Seychelles Strategy for the Prevention and Control of Non-Communicable Diseases (2016–2025) [23]. Seychelles does not have an oral health plan or policy that addresses service provision, dental public health or resources. As such, the health policy documents and regulations shape the health and oral health situation of the country. Despite the policies and strategic plans covering comprehensive health issues, the lack of inclusion of oral health appears to have a direct impact on oral health outcomes. Moreover, the barriers and opportunities that these policies create for the Seychellois population have not been fully explored and understood. This study therefore aimed to understand the factors influencing the inclusion of oral health in health policy and regulations in the Seychelles. The barriers and opportunities for this lack of inclusion are also explored. This study serves as a reference for future oral health policy development in the Seychelles.

## Methodology

This study was part of a PhD study ((BM18/3/13)) that aimed to develop a framework for oral health provision through an exploration of the factors influencing the oral health in Seychelles. A qualitative approach was adopted using document analysis and interviews as data collection strategies to allowed for a complete analysis of the research problem [24]. The interviews provided for complex textual accounts by participants and ensured for elusive factors to be identified and explored [24]. The analysis of documents involved the extraction of information from policy documents sourced from the Health Ministry archives as well as from online sources. These documents were analysed for the inclusion of oral health with regards to the number of times ‘oral health’ related text appeared in policy documents and its context.

A purposive sampling method was used because the study required rich accounts from participants who are directly related to the phenomena under study. Data collection consisted of primary and secondary sources. Staff of the upper management at the Ministry of Health and twenty public dental health staff and were invited to participate in the study. Following the invitation to interview two upper management representatives, only one participated. The dental staff included 2 dental specialists, 4 dentists, 3 dental hygienists, 2 dental laboratory staff, 5 dental surgery assistants, and 4 dental therapists. The inclusion criteria were that the staff had to have more than 5 years of continuous work experience with the exception of dental hygienists. The researcher felt that this was the minimum continuous number of years a staff needed to be working to have expert knowledge on the topic. The three dental hygienists from the first training cohort had only 3 years’ work experience at the time of data collection but were invited based on their ability to provide information pertinent to the research questions. No other higher-level representative needed to be contacted to participate as some of the dental staff interviewed were already involved in higher management meetings/decision-making and could provide rich information. The primary sources of the data consisted of individual face-to-face interviews. Whilst this study focused attention to policy and employers in the health and oral health sector, the perspectives of the patients are important and will add value to the perceptions of the health care system. This aspect is included in a follow up article. Thematic analysis was utilized to analyse the data emanating from the interviews. Thematic analysis is a method that is useful in managing, organizing and presenting qualitative research and as such facilitates reporting of experiences and meanings, and the reality of participants [24]. This method involved the process of coding, generating themes, establishing relationships and interpreting the findings. ATLAS. Ti, Version 8.1.3 (522) [25] was used as a workbench for organisational and technical support during the coding of the data and the development of themes.

Data collected through secondary data sources was obtained from the reviewing of public health policies and regulations which had a potential influence on oral health outcomes in Seychelles. As oral diseases such as, dental caries, periodontal diseases, oral cancer and facial injury/trauma were commonly observed amongst the Seychellois population, regulations, legislations, and health programs that has potential influence on these oral diseases were examined. The prevalence and treatment options of these conditions as well as oral health related information were sourced through content analysis. Data was extracted through the counting and measuring of the occurrence of oral health related statements in the documents; and through the interpretation and understanding of the extent oral health was represented in the documents. The documents analysed are listed below for ease of reference and were selected because of the common risks for oral diseases and general health conditions.

1. National Health Policy – 2016 [20].
2. Seychelles National Health Strategic Plan, 2016–2020 [22]
3. Seychelles Strategy for the Prevention and Control of Non-Communicable Diseases, 2016–2025 [23].
4. National School Nutrition Policy – 2008 [21].
5. Seychelles National Alcohol Policy – 2016 [26].
6. Tobacco Control Act – 2009 and Tobacco Control (Smoke-Free Notice) Regulations -2011 [27].
7. The Goods and Service Tax Act Regulations – 2003 [28].
8. Excise Tax (Imposition of Sugar Tax on Drinks) Regulations − 2019 [29].
9. Seychelles Trade Tax Schedule 2–2009 [30].

Ethics approval was obtained from the Biomedical Science Research Ethics Committee of the University of the Western Cape, South Africa (Ethics approval number BM18/3/13) and the Health Research and Ethics Committee (HREC) of Seychelles (Ethics approval 1807). All methods used to collect data were carried out in accordance with relevant guidelines and regulations of the University Ethics Committee as well as the Health Research and Ethics Committee (HREC) of Seychelles. Written informed consent to participate in the study was obtained from the staff of the Ministry of Health. An information sheet outlining the details and purpose of the research was provided in Creole and in English. Participants were also informed of their right to withdraw from the study at any point throughout the research process without any negative consequences. To protect the participants’ identities, all names were substituted with study identification numbers. Moreover, any words that may be related to the research participants that could lead to their recognition were substituted to ensure confidentiality. Participants were made aware prior to and at the start of the interviews that the information collected was to be used to write up a thesis and for dissemination through meetings with government officials in Seychelles as well as published in journals.

## Results

This section reports on the inclusion of oral health in key health policies and regulation in the Seychelles, the factors influencing the inclusion of oral health in such documents, including the barriers and opportunities for the lack of inclusion.

### Oral health in key public health policy and regulations

The study revealed that there was minimal representation of oral health in health policy and regulations in the Seychelles. Only 3 out of the 10 documents examined had statements about oral health. Oral health was mentioned in the following ways: oral diseases in relation to other NCDs affecting Seychelles; the reduction of dental caries as an outcome of policy implementation; and through the taxation regulations of dental products. The reason for the lack of inclusion of oral health in key health policy and regulations in the Seychelles was unclear but data collected through interviews showed that oral health was low on the health agenda. Two participants explained:

I have a feeling that dental is still not a topic where others take much care about what is a hole in the tooth. It is probably less important than a hole in your car tyre. That should somehow change… Dental is not on top of the list but rather at the bottom of the list. I mean general dental problem is not as important as a cardiac or whatsoever and that is how they are dealing with this. Dental somehow is somewhere at the end of the food chain [Staff 6].

Oral diseases are considered as NCDs nowadays which is a priority for us [Ministry of Health] as the risk factors are the same. So, we are tackling oral health through strategies for NCDs so I think it is covering [Staff 21].

The minimal attention to oral health on the health agenda was evident in the way that it usually presents as an indirect outcome of many of the health policy documents. The assumption was that the CEO of the Health Care Agency (HCA) represents the OHS at higher level meetings and should logistically bring forward the agenda of the OHS as one participant explained:

Policy which involves NCDs at time we [Ministry of Health] forget to involve dental. We forget to involve all the specialties even eyes, but we expect the CEO of HCA to speak on behalf of dental and bring to meetings as dental falls under HCA [Staff 21].

This practice was not always at the best interest of the OHS due to the Ministry of Health organogram. This is because the CEO of the HCA is responsible to manage the overall operations of all clinical services offered by the Ministry of Health and not just the OHS.

### Assessment of oral health policy

At the time of the research there was no national oral health policy and strategic plan in the Seychelles. In 1999 an oral health policy (2001–2010) was drafted but this was put on hold for reasons unknown. The vision of the policy was that within 10 years of implementation a comprehensive oral health system would be established, one that is fully integrated within general health and based on the principles of primary health care, with emphasis on prevention of oral disease and oral health promotion. At the time of the research, there was a lack of national oral surveys to establish the evolving rates of oral disease or what influenced these, neither was there water, salt fluoridation or dental sealant programmes in Seychelles. Despite water fluoridation being a recommendation in numerous oral health policies worldwide, there were mixed feelings about its implementation in Seychelles amongst health professionals. Other alternatives such as milk fluoridation were often proposed as explained in the accounts of two participants:

I do not think we should put fluoride in the water yet. I think there are a lot of things that needs to be evaluated with our system, with our water sources because [in] Seychelles there are a lot of water sources that are not centralized and we need manpower and technical workforce and equipment [Staff 16].

I will say remove the water fluoridation for kids put in milk, because in milk the calcium is good for the bones or maybe salt something which you know people will use [Staff 4].

### Policy as a catalyst for industry

The interviews indicated that there is evidence of a gap in public health policies in the Seychelles. For instance, the loop holes in some of these policies creates opportunities for commercial companies or individuals to push forward their agenda even though there may be a negative impact on oral health outcomes. These include food companies selling unhealthy food and beverages through television advertisement, newspaper and internet. This gap in the policy documents was due to other ministries formulating health-related policies without consulting the Ministry of Health, and the lack of monitoring of public policies by law enforcers. Participants’ accounts reflect this notion:

It is a policy that they [the government] have put in place and then try to get a medical justification to support it [Staff 21].

They are saying that there is a policy in school but a lot of tuck shop are selling under the counter. It is a big problem, [in] our society… You see a pick up [truck] full of packets of sweets. Three tons with packets of sweets but I am telling you it is a bit too much. The government needs to know how we are fighting. It is a fight we are losing. When you check you do your bit but the authority is doing nothing [Staff 7].

### Taxation policy

Currently in the Seychelles dental products including toothpastes are classified as cosmetic surgery whereby there is a 15% Value Added Tax (VAT) added on the products. This acted as barriers for the public to purchase and utilize them as summarized in one participant’s response:

It (taxes on dental products) is exorbitant. It is like you say there are taxes on sanitary pads for women. It is the same way for dental products. All these dental products, a floss, a basic should not be costing that much because it is all about prevention. So, if you have a floss which is more affordable to a person who earns 5000 rupees he is definitely not going to be happy to spend 50 bucks on buying a dental floss and then you tell them to use the length of your arm so that there is not reuse between several teeth. So, it is not a question of if you want to make it more accessible to your people you reduce the taxes for these dental products. Women need sanitary pads. People need their floss, they need their fluoridated toothpaste, and they need their soft toothbrush. If all these things were affordable I think people would have a better oral hygiene care at least and because that is the basis for everything [Staff 9].

### Provision in health budgets

Allocation of resources for the proper functioning of oral health services was partly influenced by the absence of an oral health policy to guide service delivery. Allocation of funding was often based on the priority of the project or the activity level of a unit at the Ministry of Health which often involved extensive paper work with justifications. This resulted in the budget being primarily directed towards curative care (management of oral diseases and restoring oral function), which has consequently resulted in a reduction in oral health prevention programmes. The OHS has been implementing a few programs in the population aimed at improving the oral health of specific population groups for example the Maternal Child Oral Health Programme, the oral health - Antenatal Programme; and the school oral health programme. However, program coverage is low due to the bureaucracies to access funding for prevention. This is reflected in the following participants’ response:

Well dental… gets a budget for consumables of materials, I think over the past 20 years it has not change… Prevention is at the level of the Ministry or HCA [Health Care Agency]. There are departments that do prevention… dental does not form part of the different departments [Staff 16].

Money is allocated for those [units in health] that make more noises. Even in parliament those that makes more noises for their district get more money, which shows you the importance of advocating. If you are not advocating and staying quiet and on the other side people are advocating more powerful, it is normal who gets the funds [Staff 21].

To secure the budget [Oral Health Services] has to work with the different units to bring forward projects… We [Oral Health Services] need to justify when we ask…. We get in the end. It is not easy but you get [Staff 16].

### Recruitment of staff

There is no Dental school in the Seychelles. Dentists and dental specialists are trained internationally. However, as a once off project, only two cohorts of Dental hygienists were trained locally. In addition, a lack of attractive salary packages for the recruitment and retention of local primary oral health professionals to address the burden of disease often had an influence on service delivery in public dental facilities. As such the majority of dentists and dental specialists working in Seychelles are foreigners. They are not familiar with the context of the Seychelles, the challenges within the health care system, the language and the culture and who have different languages and backgrounds. High costs are also incurred to recruit international dentists and whose attributes are not verified by Seychelles Medical and Dental Council. The recruitment process was experienced as challenges to the oral health system and particularly for effective patient care. This view is captured in the accounts of two participants:

Our [OHS] struggle is more on dentists because we are recruiting from overseas three quarter of the time and this cost a lot of money, a lot of procedures and in terms of quality we get what we can afford… Three quarters of dentists are not Seychellois so there is the communication. It is the love which I think is different from Seychellois who is treating his/her own nation and a foreigner, it is a pity that we do not have a lot [of Seychellois] [Staff 16].

We [OHS] simply need enough staff and engaged staff motivated… if the salary is alright then you have motivated people and you can employ the dentists from well reputed universities which are trying to make a good job and fillings that last not only 6 months but maybe 5 years… Patients who comes in the district clinic for the same problem 10 times in 5 years, this is a waste of resources, money and whatsoever [Staff 6].

### School curricular

The lack of inclusion of oral health in the school curriculum was identified as a contributing factor to poor oral health outcomes in the Seychelles. The assumption was that through the implementation of activities such as nutrition education, school tuck shop guidelines, and by encouraging students to drink water there will be a reduction in certain non-communicable diseases, dental caries included. The insufficient representation of oral health in school curriculum resulted in minimum exposure to oral health messages, and services which are needed to inform oral health choices and the adoption of appropriate oral health related behavior. One participant explained:

I am thinking there is not enough follow up and also [no] program in the school. It is lacking because in the past we did a lot. There was health promotion, we went in the school, we went for talks to educate children on mouth health [and] on the way to brush but that has reduced. So, I think it plays a role [Staff 15].

The implications of structure of the health care system, the policies and regulations as well as how the school curriculum influences oral health service delivery and by extension oral health outcomes will be discussed in this paper.

## Discussion

The lack oral health policies and its inclusion in public policies, has impacted the country in several ways. Firstly, the manner in which the organisational structure of the Ministry of Health of the Seychelles is designed often impacts on the inclusion of oral health in relevant policy and on the delivery of oral care. The structure is influenced by having insufficient active representation of oral health in the Ministry, where policy and strategic documents are developed. As a consequence, in Seychelles there is an inevitable absence of appropriate oral health policies or strategic plans at a macro level accompanied by poor allocation of funding which are important to implement and steer oral health initiatives in order to reduce the burden of oral diseases [1]. Moreover, this challenge of developing policies is being exacerbated by having non-dental professionals advocating on behalf of oral health professionals for the needs of the country. As such informed decisions about oral health provision may be compromised. Policy development, across all sectors, lies on the buildup of collaboration and use of power to facilitate change by individuals implicated in the policy process [1].

Secondly, public health policies or guiding documents in Seychelles prioritise and focus on a range of health issues other than oral health, contributing to the lack of inclusive strategic plans that should be filtered to the district level. Similar to many countries, it can be argued that policies in the Seychelles have given more attention to general health care strategies that do not support the management of the burden of oral diseases. A more feasible approach would lie in developing and implementing a fully integrated and synchronised National Health and oral health plan or policy using a common risk factor approach [1]. The current exclusion of oral health combined with the lack of government support in providing guiding documents and limited funding, may be indicative that oral health will unlikely be a priority nor be included in future health reform in the Seychelles unless change is effected at government level. This deficit can be seen in the way that oral disease prevention has been lacking within the NCDs policy agenda at a national level in Seychelles. As such, it has impacted how oral health care in the current study was potentially accessed by the population, in how it has influenced oral health behaviour and by extension in how it has the affected oral health outcomes of the public. The inequitable access to oral health care and the oral disease burden is a consequence of the absence of an oral health policy. It has further impacted on the comprehensive management of patients, coverage of oral health promotion activities, as well as the level of oral health literacy of the community.

Thirdly, the allocation of human resources to meet the needs of the Seychellois communities would have been better managed if it would have typically been part of an oral health policy. Studies [31, 32] impress that the training and recruitment of adequate oral health professionals to compensate for the inequitable distribution of oral health services is crucial to reduce inequity in oral health outcomes. The lack of oral health has impacted on how primary oral health manpower are recruited, distributed and respond to the burden of diseases particularly according to the specific needs of the population. Such includes the timeliness and availability of clinical preventive and curative dental care, and the accessibility and coverage of community-oriented prevention and health promotion [30]. Furthermore, recruiting dental professionals who may not be fully equipped or skilled to address the Seychelles needs has the potential to impact on quality of care offered to the general public. This problem may lead to patients not trusting the oral health system which in turn has the potential to influence the delay in accessing dental care leading to disease progression and costlier intervention.

Fourthly, the current study found that public policy content and implementation were not always aligned in support of oral disease prevention in Seychelles. The loop holes in some public policies creates opportunities for commercial companies or individuals to sell their products even though there may be a negative impact on oral health behavior. For example, media advertising has the potential to influence the population’s opinion and practices. Prohibition in the advertising of alcohol is limited to television, whilst there is no prohibition on the advertising of unhealthy food and beverages. In addition, the 15% VAT placed on mouth care products in Seychelles is influencing the choices people make regarding their oral hygiene care leading to the unaffordability of appropriate dental products to support prevention initiatives. In the socioeconomically disadvantaged groups or households this challenge will be more apparent. The focus should shift towards public policies which have strategies to reduce the oral health disparities, for example, through the importation of low-cost fluoridated toothpastes which is fundamental to improve oral health outcomes of the population of Seychelles [33, 34].

Lastly, as is the case in South Africa, school health care is advocated as a valuable context and setting for increasing oral health literacy and for the implementation of oral health promotion activities [6, 35]. In the Seychelles, the lack of integration of oral health in certain school policies and strategic documents also impacted On the oral health outcomes . For example, the lack of integration of oral health content in school curriculum implied that children are not exposed with oral health information at an early stage. This deficit will have an impact on how oral health behaviours of children are developed and in turn influence oral health outcomes of the Seychelles population. The inclusion of oral health in school education is deemed crucial for the adoption of appropriate habits conducive for good oral health at an early age [35–37].

Oral health problems can be prevented by health agencies that develop and implement regulations and legislation as part of an integrated health plan and one that favours a supportive environment for achieving positive oral health outcomes. However, oral health will not advance as a planned initiative if it stays detached from health policy or is excluded from the multi-sectoral agenda for health promotion [35, 38]. On the contrary, as has been advocated for decades, health policies ought to be reoriented to include oral health using social and dental approaches to assess needs and to guide the incorporation of oral health into the common risk factor strategy for health promotion [1, 9, 39, 40].

A national oral health policy is meant to guide oral health planning, distribution of oral health services and strategies aimed at addressing the oral health burden and disparities [41]. Instituting policy will improve the quality of life and eradicate health inequalities through enabling partnerships among individuals, health and oral health service providers, communities, and policymakers at all levels of society and by taking advantage of current plans. National oral surveys must also be conducted periodically and used as evidence upon which to lobby for change in the prevention of Oral diseases. As a WHO member state, it is thus imperative for Seychelles to implement appropriate policies that address the determinants of poor oral health in the Seychelles, so as to ensure that oral health is available to all without hardship by 2030 [1].

## Conclusion

This study aimed to understand the factors influencing the inclusion of oral health in health and public policy and regulations in the Seychelles. The barriers and opportunities for inclusion / non-inclusion as well as the impact thereof were explored. Insights were provided on how oral health is contextualised in the Seychelles and how public policy and strategic documents influences the oral health outcomes. There is fragmentation in how the health and oral health agendas are shaped coupled with a lack of involvement at various levels to address the latter. The deficit of integrated health and oral health policies or strategies impedes on the direction to address the oral disease burden in the country. Oral health needs to be integrated in all relevant policies and public health programmes as part of the broader national NCDs in Seychelles in order to design strategies aimed to reduce the incidence of oral diseases in the population.

## Data Availability

The datasets generated and/or analysed during the current study are included in this published article. A comprehensive dataset is also available in the UWC Scholar – ETD Repository [https://etd.uwc.ac.za/handle/11394/8756].
